# Supplementary material for: Antioxidant Activity of Different Hop (Humulus lupulus L.) Genotypes
Source: Plants (Basel). 2023 Sep 29;12(19):3436. doi: 10.3390/plants12193436 (PMC10575397; doi:10.3390/plants12193436)
Supplement: Supplementary file 1 [file plants-12-03436-s001.zip › plants-2621186-supplementary.pdf]

Plants

Supplementary Material

Antioxidant Activity of Different Hop (*Humulus lupulus L.*) Genotypes

*Zala Kolenc[1, 3,4], Tamara Hribernik[2], Tomaž Langerholc[4], Maša Pintarič[4], Maja Prevolnik Povše[5] and Urban Bren[1,3,6] **

[1] *Laboratory of Physical Chemistry and Chemical Thermodynamics, Faculty of Chemistry and Chemical Engi-neering, University of Maribor, Smetanova ulica 17, SI-2000 Maribor, Slovenia; zala.kolenc@um.si (Z.K.)*
[2] *Department of Lifestock Breeding and Nutrition, University of Maribor, Pivola 10, SI-2311 Hoče, Slovenia; tamara.hribernik1996@gmail.com (T.H.)*
[3] *Department of Applied Natural Sciences, Faculty of Mathematics, Natural Sciences and Information Technologies, University of Primorska, Glagoljaška ulica 8, SI-6000 Koper, Slovenia; urban.bren@um.si (U.B.)*
[4] *Department of Microbiology, Biochemistry, Molecular Biology and Biotechnology, Faculty of Agriculture and Life Sciences, University of Maribor, Pivola 10, SI-2311 Hoče, Slovenia; tomaz.langerholc@um.si (T.L.); ma-sa.pintaric@um.si (M.P.)*
[5] *Department of Animal Science, Faculty of Agriculture and Life Sciences, University of Maribor, Pivola 10, SI-2311 Hoče, Slovenia; maja.prevolnik@um.si (M.P.P.)*
[6] *Institute of Environmental Protection and Sensors, Beloruska ulica 7, SI-2000 Maribor, Slovenia*
* *Correspondence: urban.bren@um.si, Tel.: +386-2-229-4421*

* Correspondening Author
E-mail addres: urban.bren@um.si
tel.: +386-2-229-4421.

Table S1: FRAP, ORAC and IA potential values.

Samples/Measurement	FRAP (μmol TE/g DM)	ORAC (μmol TE/g DM)	IA (mmol TE/g DM)
**Hop genotypes**			
Aurora	68.7±1.3	1909.1±53.9	88.6±16.0
Savinjski golding	101.6±2.2	1695.6±102.1	52.7±11.5
Styrian Wolf	68.6±0.7	1686.7±67.4	83.3±24.3
Styrian Dragon	70.6±1.8	1592.7±49.0	78.4±18.3
Styrian Eureka	63.5±0.5	1910.7±73.8	79.5±25.5
Styrian Fox	69.2±2.9	1722.3±31.6	81.8±30.1
Styrian Eagle	70.5±0.4	1653.7±69.4	112.4±8.1
Chocotsu No.17 S168 Japan	82.0±1.4	1496.7±29.0	73.3±1.5
Nugget (USA) S222	75.1±1.1	1723.8±39.9	118.0±31.8
Belgium S367 P157	86.4±1.8	1735.8±21.1	88.7±26.6
Dekorativny (Russia) S248	90.0±0.5	1686.9±77.2	106.2±29.3
Early promise (England) S68	85.9±2.1	1544.1±39.1	78.7±16.0
Canada P169 S369	82.8±2.1	1588.9±43.5	92.4±21.5
Caucasus S353 P15	97.2±0.6	1069.4±34.3	65.0±10.1
**Hop purified extracts**			
αβ - AF	121.3±0.7	861.6±60.6	68.6±28.0
β - AF	79.8±4.3	992.7±56.5	150.4±32.1

The average ± standard deviation is presented.
